# Defining the organizational structure of dopamine and muscarninic acetylcholine receptors

**DOI:** 10.1186/2193-1801-4-S1-L7

**Published:** 2015-06-12

**Authors:** Graeme Milligan, María José Varela Liste, Gianluigi Caltabiano Caltabiano, Elisa Alvarez-Curto, Sara Marsango

**Affiliations:** University of Glasgow, UK; Universitat Autònoma de Barcelona, Spain

**Keywords:** Dopmaine, acetylcholine receptor structure

G protein-coupled receptors, including the M3 muscarinic acetylcholine receptor and the dopamine D3 receptor, can form homo-oligomers. However, the basis of these interactions and the overall organizational structure of such oligomers are poorly understood. Combinations of site-directed mutagenesis and homogenous time-resolved FRET studies that assessed interactions between receptor protomers at the surface of transfected cells indicated important contributions of regions of transmembrane domains I, IV, V, VI and VII, as well as intracellular helix VIII, to the overall organization. Molecular modelling studies based on both these results and an X-ray structure of the inactive state of the M3 receptor bound by the antagonist/inverse agonist tiotropium were then employed. The results could be accommodated fully by models in which a proportion of the cell surface M3 receptor population is a tetramer with rhombic, but not linear, orientation. This is consistent with previous studies based on spectrally-resolved, multi-photon FRET. Modelling studies suggest, furthermore, an important role for molecules of cholesterol at the dimer + dimer interface of the tetramer, consistent with the presence of cholesterol at key locations in many G protein-coupled receptor crystal structures. Mutants that displayed disrupted quaternary organization were often poorly expressed and showed immature N-glycosylation. Sustained treatment of cells expressing such mutants with the muscarinic receptor inverse agonist atropine increased cellular levels and restored both cell surface delivery and quaternary organization to many of the mutants. These observations suggest that organization as a tetramer may occur before plasma membrane delivery and may be a key step in cellular quality control assessment.

